# Hairpin loop to hairpin loop: a full-length assembly of the ASFV genome using Oxford Nanopore long-read sequencing

**DOI:** 10.3389/fmicb.2025.1615977

**Published:** 2025-08-08

**Authors:** Poompat Phadphon, Chutima Sonthirod, Theeradej Thaweerattanasinp, Jeremy R. Shearman, Sonicha U-thoomporn, Janya Saenboonrueng, Asawin Wanitchang, Sithichoke Tangphatsornruang, Anan Jongkaewwattana, Wirulda Pootakham

**Affiliations:** ^1^Genomic Research Team, National Omics Center, National Center for Genetic Engineering and Biotechnology (BIOTEC), National Science and Technology Development Agency (NSTDA), Pathum Thani, Thailand; ^2^Virology and Vaccine Technology Research Team, National Center for Genetic Engineering and Biotechnology (BIOTEC), National Science and Technology Development Agency (NSTDA), Pathum Thani, Thailand

**Keywords:** hairpin loop, MA-104 cells, African swine fever virus, ASFV, adaptation

## Abstract

Short-read assembly of the African swine fever virus (ASFV) genome is challenging due to the presence of inverted terminal repeat (ITR) and hairpin loop sequences, which often cause ambiguity in contig reconstruction. In this study, we employed Oxford Nanopore long-read sequencing to assemble a full-length ASFV genome from passage 50 of an ASFV strain adapted to MA-104 cells. We identified duplicated reverse complementary reads from the ITR and hairpin loop regions, which, if not properly analyzed, could lead to an inaccurate assembly that falsely represents these complex regions. Our findings highlight the power of long-read sequencing for resolving complex viral genomes and reveal potential challenges for other viruses with similar terminal structures.

## 1 Introduction

African swine fever virus (ASFV) is a highly pathogenic virus that produces a deadly hemorrhagic disease in both domestic and feral pigs, with extremely high mortality rates. Since 2007, ASFV has spread across several countries in Europe and Asia, wiping out hundreds of millions of pigs (Dixon et al., 2019). This transboundary virus causes severe economic losses and poses a significant threat to the global pig industry and food security. Despite extensive research, no effective vaccine or treatment is currently approved for ASFV. Moreover, the complete molecular mechanisms underlying ASFV pathogenicity remain to be fully elucidated.

ASFV is a large, complex DNA virus belonging to the *Asfarviridae* family. Its genome structure has been described as linear, double-stranded DNA of approximately 170–190 kb that encodes 151–167 open reading frames (ORFs) (Dixon et al., 2013). The ASFV genome can be divided into three major regions: the central conserved region (CCR, ∼125 kb), which is flanked by the left variable region (LVR, ∼40 kb) and the right variable region (RVR, ∼20 kb). Genes in the CCR play significant roles in viral replication, assembly, and immune evasion. Most of the genes in the LVR and RVR are members of five multigene families (MGF), including MGF 100, 110, 300, 360 and 505, which are involved in virus replication, cell and host tropism, immune evasion, and virus virulence (Chapman et al., 2008; Dixon et al., 2013).

Both ends of the linear double-stranded genome contain inverted terminal repeats (ITRs) and hairpin loops, which play important roles in genome stability, replication, and packaging (Gonzalez et al., 1986; Dixon et al., 2013). These regions of the genome are difficult to assemble when using only short-read sequencing data (Forth et al., 2019). The BA71V genome assembly, the only genome with complete ITR and hairpin loop sequences, revealed four open reading frames (ORFs) within the ITRs (Yanez et al., 1995). This emphasizes the importance of a full-length ASFV genome assembly with complete ITRs and hairpin loop sequences. The limitations of short-read sequencing highlight the need for long-read sequencing such as Oxford Nanopore technology (Oxford Nanopore Technologies, Oxford, United Kingdom). Long reads (> 10 kb) aid in creating complete, high-quality ASFV genome assemblies (Scarano et al., 2024), and resolving the ambiguity in assembling the ITR and hairpin loop regions. In this study, we aimed to generate a full-length ASFV genome assembly of Chonburi_2024_209-MA that includes intact terminal hairpin structures using Oxford Nanopore long-read sequencing, and to identify potential methodological challenges specific to sequencing genomes with terminal hairpin loops.

## 2 Material and methods

Aiming to get a full-length ASFV genome, we employed Oxford Nanopore Technologies (ONT) to sequence the genome of a cell culture-adapted ASFV originally isolated from infected Large White pigs in Phanat Nikhom District, Chonburi, Thailand in August 2023. The isolated ASFV was cultured in MA-104 cells (ATCC CRL-2378.1) in DMEM (Cytiva; Cat# SH30243.02) with 10% fetal bovine serum (FBS; Sigma; Cat# F7524) and was serially passaged until passage 50. Details of ASFV isolation and passaging can be found in a previous study (Thaweerattanasinp et al., 2024). At passage 50, the cell suspension was centrifuged at 5,000 × *g* for 10 min at 4°C. Cell supernatant was then harvested, filtered through 0.45-μm syringe filters, and concentrated to a volume of 20 mL using Amicon Ultra centrifugal filters (Merck; Cat# UFC9100). The resulting supernatant was ultracentrifuged at 150,000 × *g* for 3 h at 4°C. The supernatant was then carefully decanted into a waste container, while the pellet was resuspended in 1 mL of PBS. ASFV DNA was extracted from the resuspended pellet using the High Pure Viral Nucleic Acid Kit (Roche; Cat# 11858874001) according to the manufacturer’s instructions. This cell culture-adapted ASFV isolate was named Chonburi_ 2021_209-MA.

The extracted DNA was sequenced with both long-read and short-read technologies. Oxford Nanopore Technologies was applied for long-read sequencing. The PCR-free library was prepared from 250 ng of high molecular weight genomic DNA using a 1D Native Barcoding Kit 24 V14 (SQK-NBD114.24; Oxford Nanopore Technologies, Oxford, United Kingdom). The library was loaded onto a R10.4.1 flow cell and ran for 12 h on a MinION Mk1C sequencer. Regarding short-read sequencing, genomic DNA was used for library preparation with MGIEasy FS DNA Library Prep Set (MGI Tech, Shenzhen, China) according to the manufacturer’s instructions. The library was sequenced using DNASEQ-G400 (150-bp paired-end reads).

Oxford Nanopore read base calling was done using Guppy v6.3.8 (Oxford Nanopore Technologies, Oxford, United Kingdom). Oxford Nanopore reads were mapped on ASFV Georgia 2007/1 (GCF_003047755.2) using minimap2 v2.26 (Li, 2018). Mapped reads were extracted using SAMtools v1.17 (Danecek et al., 2021). Since Oxford Nanopore reads are prone to high error rates, we performed read correction, which denoised reads by generating consensus sequences of overlapping reads, and trimming using CANU v2.2 (Koren et al., 2017) prior to assembly with the same software. The corrected and trimmed Oxford Nanopore reads and MGI short reads were mapped onto the draft Chonburi_2024_209-MA assembly using minimap2 and Bowtie2 v2.5.1 (Langmead and Salzberg, 2012), respectively. Read depth supporting every nucleotide of the draft assembly was called using SAMtools. MUMmer4 (Marcais et al., 2018) was used to generate an alignment dot plot between the draft assembly and Georgia2007/1 genome.

To get a complete full-length genome assembly, we first determined the hairpin loop sequences at both termini of the draft assembly. The terminal DRC sequences extending beyond the hairpin loop sequences have been trimmed off. The trimmed assembly was polished with short reads using Pilon v1.24 (Walker et al., 2014). To maximize the accuracy of the assembly, a polishing step was repeated until there were no additional nucleotide changes. The trimmed and polished assembly, so called complete full-length Chonburi_2024_209-MA, was subsequently annotated using Prokka v1.14.6 (Seemann, 2014).

To investigate genetic variations, comparative analyses were performed. Complete full-length Chonburi_2024_209-MA assembly, Georgia2007/1, the first reported genotype II ASFV in China: Pig/HLJ/2018 (GCA_004338215), the first hairpin-to-hairpin ASFV genome: BA71V (GCF_000858485), and ONT-based HK_NT_202103 (GCA_030515915) were included in the analyses. To illustrate the advantage of long-read assembly, we constructed short-read assembly of Chonburi_2024_209-MA, so called Chonburi_2024_209-MA short-read assembly and included it in the comparative analyses. In short-read assembly, short reads were mapped on the draft Chonburi_2024_209-MA assembly with Bowtie2. Mapped reads were extracted using SAMtools and assembled with SPAdes v3.15.4 (Prjibelski et al., 2020).

Six ASFV genomes in total were aligned with MAFFT v7.525. To investigate structural variation among genomes, the boundaries of alignment blocks in each genome were extracted and visualized. We also assessed nucleotide-level variation i.e., SNPs, and indels. However, Chonburi_2024_209-MA short-read assembly and BA71V were excluded from the analysis. The alignment revealed no difference between the complete full-length Chonburi_2024_209-MA assembly and Chonburi_2024_209-MA short-read assembly. Meanwhile, BA71V, a genotype I ASFV, exhibited an unnecessarily high number of variations, making it unsuitable for comparative analysis in this level. The genomes of complete full-length Chonburi_2024_209-MA, Pig/HLJ/2018, and HK_NT_202103 were mapped to Georgia2007/1. Variants were called against a reference genome Georgia2007/1 using BCFtools v1.22 (Li, 2011). The effects of the variants on coding genes were assessed with SnpEff v5.2 (Cingolani et al., 2012).

## 3 Results

### 3.1 Oxford Nanopore long-read assembly

**FIGURE 1:**
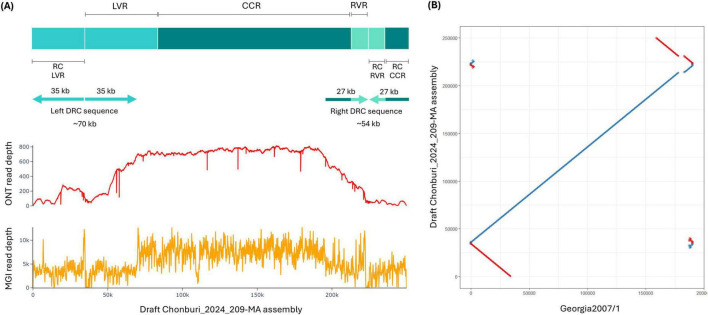
A draft Chonburi_2024_209-MA assembly based on Oxford Nanopore reads. **(A)** A draft Chonburi_2024_209-MA assembly diagram with terminal duplicated reverse complementary (DRC) sequences indicated with arrows and ONT (above) and MGI (below) read depth plots. **(B)** Dot plot of the draft Chonburi_2024_209-MA assembly against Georgia 2007/1. RC: reverse complement.

A total of 20,413 corrected and trimmed reads yielded 121 Mb of sequencing data. This provided a theoretical depth of 637× of a 190 kb genome, indicating a robust dataset for a high-confidence and complete genome assembly. Read lengths ranged from 1,000 to 73,763 kb with a mean length of ∼5,937 bases (Supplementary Figure 1). Our draft Chonburi_2024_209-MA assembly based on Oxford Nanopore reads was 249,904 bp in length. It is larger than published ASFV genomes, which in general are 170–193 kb (Dixon et al., 2013). Compared to 190,584 bp Georgia 2007/1, our draft assembly exhibits duplicated reverse complementary (DRC) sequences at both termini of the genome (Figure 1), approximately 35 and 27 kb at 5′ and 3′ termini, respectively, reminiscent of the assembly output from a cyclic genome. The reverse complement (RC) sequence of left DRC contains ∼35 kb reverse complement sequence of 48 kb LVR, while the reverse complement sequence of right DRC covered the full length (∼10 kb) reverse complement of RVR and included reverse complement of ∼17 kb of the CCR sequence (Figure 1A). An alignment dot plot between our draft Chonburi_2024_209-MA assembly and Georgia 2007/1 genome not only showed the DRC sequences but also indicated a deletion of the draft Chonburi_2024_209-MA assembly compared to Georgia 2007/1 genome (Figure 1B). When mapping the corrected and trimmed Oxford Nanopore reads against the draft assembly, there were many individual reads that spanned across the center of each DRC sequence, supporting the existence of these sequences. However, the read depth of the left and right DRC sequences was lower than the depth of the majority of the sequence (Figure 1A). The main sequence showed a mean read depth of 686×, while the left and right DRC sequences showed mean read depths of 353× and 334×, respectively (Figure 1A). Short-read mapping revealed a similar coverage pattern with 8801× at the main sequence and 3450× and 2857× at left and right DRC sequences, respectively (Figure 1A).

### 3.2 Hairpin loop investigation

**FIGURE 2:**
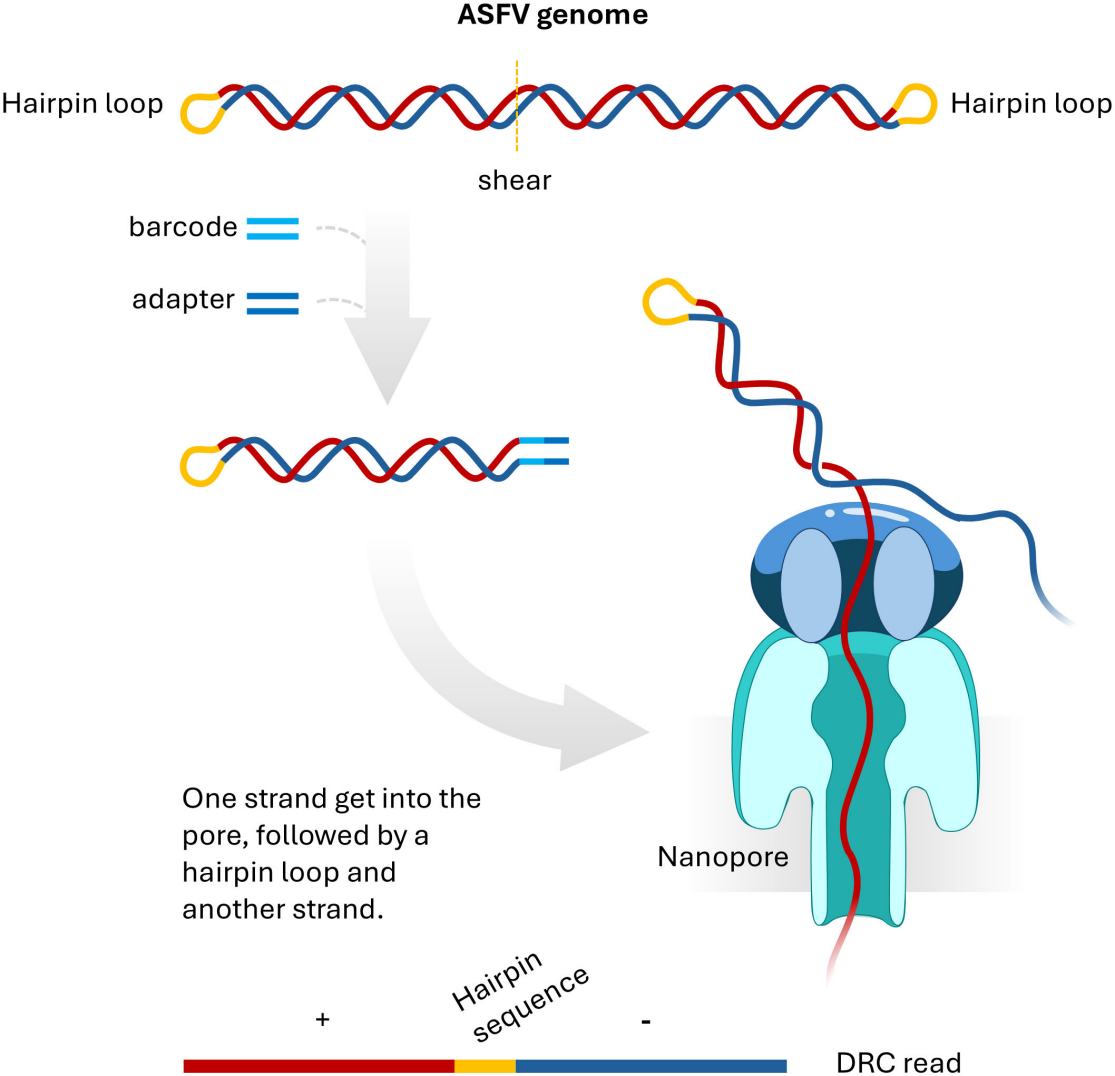
An Oxford Nanopore sequencing paradigm of double-stranded ASFV DNA fragment with a terminal hairpin loop. The DNA fragment is ligated with barcode and adapter. When running sequencing, one strand goes into a pore, followed by hairpin loop and another strand connected to the hairpin loop. This event generates a duplicated reverse complementary (DRC) read.

The ASFV genome structure has been described as a linear double-stranded DNA molecule. At both termini of the genome, there are short sequences that covalently link the two complementary strands and form hairpin loop structures (Dixon et al., 2013). Essentially, this means that denaturing the ASFV genome yields a cyclic single strand of DNA, which has important implications for sequencing. Normally, during library preparation for 1D Oxford Nanopore sequencing, double-stranded barcodes and adapters are ligated to double-stranded DNA fragments. Adapter-ligated double-stranded DNA template is unwound during sequencing and only one strand gets sequenced (van Dijk et al., 2023). It is likely that the barcode and adapter was ligated to one end of the double stranded DNA fragment containing the hairpin loop. When such a molecule passes through a nanopore, the sequencer reads both strands connected by the hairpin, resulting in reads containing a sequence followed by its reverse complement to produce DRC reads (Figure 2).

**FIGURE 3:**
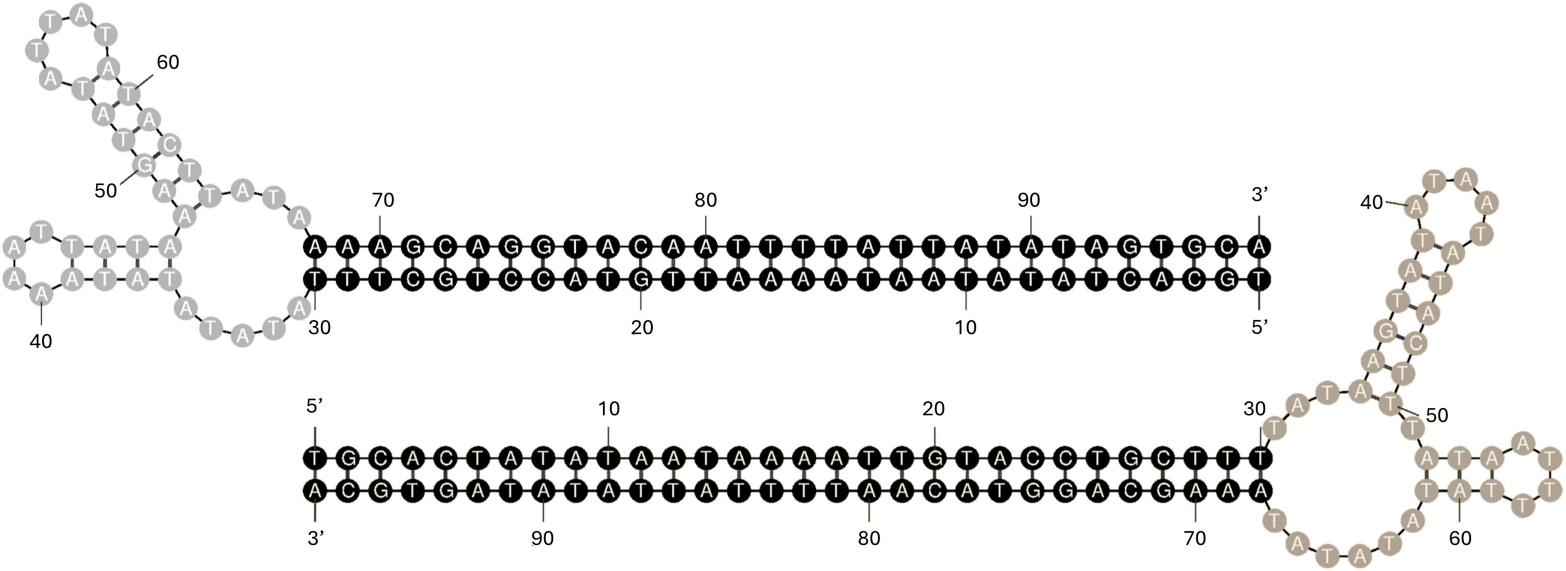
Front-terminal (above) and back-terminal (below) hairpin loop structures constructed from 97 bp-trimmed DRC reads, with the minimal free energy of –24.83 kcal/mol.

If we indeed sequenced double stranded DNA containing the hairpin, the hairpin loop sequence would be approximately at the middle of the DRC read and one half of the sequence would be the reverse complement of the other. To investigate this hypothesis, the draft assembly was cut to create front-terminal and back-terminal DRC sequences, about 70 and 50 kb in length with the hairpin loop sequence at the middle. Oxford Nanopore reads were mapped against the terminal DRC sequences. Read mapping visualization indicated reads mapped symmetrically around the center of the front-terminal and back-terminal DRC sequences with a read depth of 47× and 32×, respectively, at MAPQ 30 (Supplementary Figure 2). The mapped reads were extracted, aligned, and trimmed to approximately 97 bases in length, such that 30 bases spanned to the left and right with the potential hairpin sequence (37 nt) in the middle. The trimmed reads were then used to analyze potential hairpin loop formation using mFold web server^1^ (Zuker, 2003) with a folding temperature of 37°C and Na^+^ and Mg^2+^ concentration of 140 and 2.5 mM following DMEM component description. The trimmed reads were able to spontaneously form a hairpin loop with 37-nucleotide AT-rich sequence (Figure 3) with a minimal free energy (SantaLucia, 1998) of −24.83 kcal/mol. This supports the hypothesis that the DRC sequence reads are the result of the hairpin loop termini of the ASFV genome.

### 3.3 Complete full-length genome assembly

**FIGURE 4:**
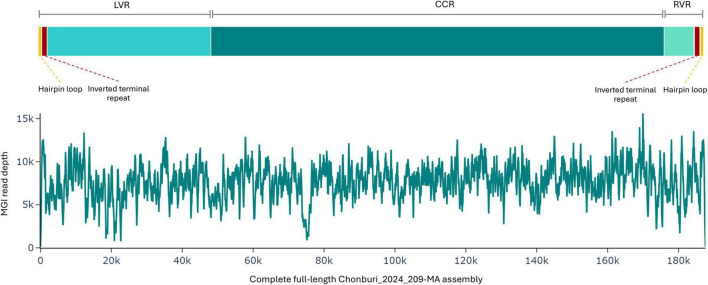
The MGI read depth of the Chonburi_2024_209-MA assembly after trimming off the terminal duplicated reverse complementary sequences.

To get a complete full-length Chonburi_2024_209-MA, the terminal DRC sequences extending beyond 37-nucleotide hairpin loop sequences of the draft assembly were trimmed off. The trimmed assembly was polished with short reads. The complete full-lenght Chonburi_2024_209-MA is 187,581 bp in length, which consists of 5′ hairpin loop sequences, LVR, CCR, RVR, and 3′ hairpin loop sequence (Figure 4). Short-read sequence depth is more evenly distributed across this version of the genome (Figure 4). The 37-nucleotide hairpin loop sequence is ATATATATAAAATTATAAAGTATATTATATACTTATA for the 5′ terminus and its reverse complement sequence is present at the 3′ terminus. The ITRs cover 2,205 nucleotides after 5′ and before 3′ hairpin loop sequences. Our ASFV genome encodes 186 genes (Supplementary Figure 3), 19 of which produce proteins smaller than 74 amino acids. These open reading frames (ORFs) are associated with various functional categories, involved in host-cell interactions, structural components, enzymatic activity, nucleotide metabolism, DNA replication and repair, and mRNA transcription of several unknown functions. Notably, both ITRs included DP60R and ACD_01990, suggesting that they may play a role in viral replication or genome stability. The complete full-length Chonburi_2024_209-MA assembly was submitted to GenBank and assigned an accession number PV339939.

### 3.4 Comparative analyses

**FIGURE 5:**
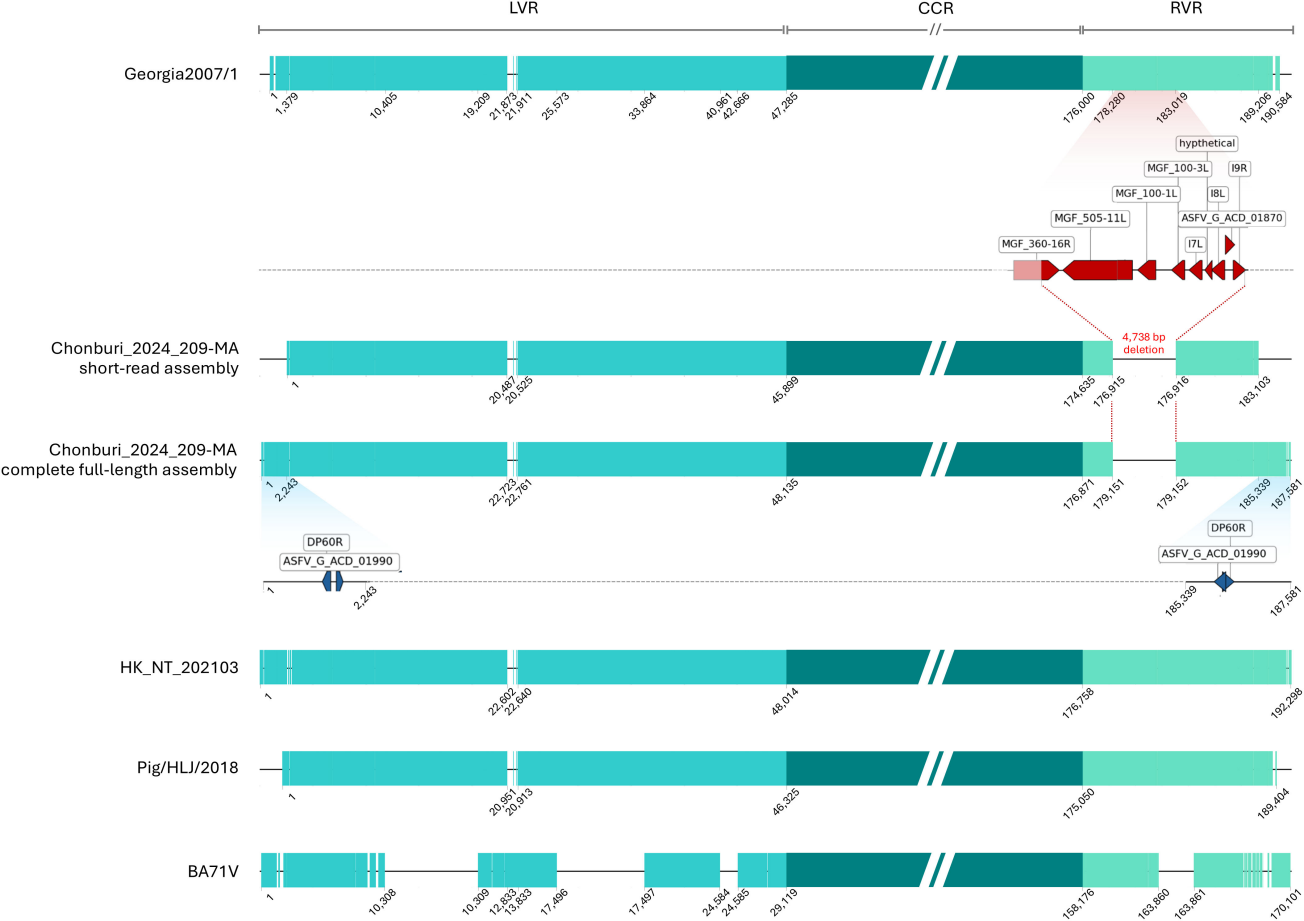
The multiple alignment blocks of six ASFV genomes, with start and end positions for each block in each genome. The 4.7 kb deletion of Chonburi_2024_209-MA assemblies compared to Georgia2007/1 was illustrated.

The multiple sequence alignment demonstrates the superiority of long-read assemblies (complete full-length Chonburi_2024_209-MA and HK_NT_202103 assemblies) that can provide more complete genome coverage than short-read assemblies (Chonburi_2024_209-MA short-read assembly, Georgia2007/1) and other techniques (a segmentation PCR of 2,400 bp in length and genome walking for Pig/HLJ/2018). Multiple alignment blocks with start and end positions of each block are shown in Figure 5. Compared to the complete full-length Chonburi_2024_209-MA assembly, Chonburi_2024_209-MA short-read assembly is shorter but shows no nucleotide differences. This highlights the high quality and accuracy of the complete full-length Chonburi_2024_209-MA assembly. The Chonburi_2024_209-MA short-read assembly is 183,103 bp in length and, when aligned to the complete genome, corresponds to the region spanning from nucleotide positions 2,243 to 185,339 (Figure 5). Therefore, the Chonburi_2024_209-MA short-read assembly does not contain ITRs, leading to the absence of two genes–DP60R and ASFV_G_ACD_01990, as well as the hairpin loop sequences at both termini. This highlights the advantage of long-read assemblies, which are more effective at resolving repeat-rich regions compared to short-read assemblies. Both Chonburi_2024_209-MA complete full-length assembly and short-read assembly indicated the deletion of 4,738 bp at position 178,281 to 183,018 compared to Georgia2007/1. This deletion covers MGF360-16R, MGF505-11L, MGF100-1L, MGF100-3L, I7L, a hypothetical gene, I8L, ACD_01870, and I9R genes (Figure 4).

Considering the hairpin loop sequence-containing genomes, complete full-length Chonburi_2024_209-MA assembly, BA71V, and the ONT-based HK_NT202103, we noticed that HK_NT_202103 genome, 192,298 bp in length, contains potential hairpin loop sequences near both termini. The multiple sequence alignment reveals 159 and 88 bp sequences extending beyond the potential hairpin loop at the 5′ and 3′ termini, respectively. Like the DRC sequences, these terminal extensions are capable of folding and forming complementary base pairing with adjacent regions.

Variant calling of three genotype II ASFV genomes, complete full-length Chonburi_2024_209-MA, Pig/HLJ/2018, and HK_NT_202103, against a reference genome Georgia2007/1 identified 123 single nucleotide polymorphisms (SNPs) and 14 insertions/deletions (indels), affecting 33 coding genes (Supplementary Table 1). Functional annotation of these variants predicted that 64.23% would cause missense mutations, 24.82% synonymous substitutions, and 10.95% would result in frameshifts or nonsense mutations. There were 3 genes showing a high density of variants, MGF_360-1La, MGF_505-2R, and MGF_505-3R. The MGF_360-1La gene exhibited 61 variant loci, which resulted from a distinct sequence of HK_NT_202103, while the others showed similar sequences. There were 19 and 21 variant loci identified in MGF_505-2R and MGF_505-3R, respectively, where the complete full-length Chonburi_2024_209-MA genome was unique from the others. Among the remaining 36 variant loci, 32 loci showed differences between Chonburi_2024_209-MA and Georgia2007/1. Of these 32 loci, the variants of 8 loci were shared with at least one other genome, while 24 loci were unique to the Chonburi_2024_209-MA genome.

Surprisingly, the unique 19 variant loci in MGF_505-2R and 21 loci in MGF_505-3R of the complete-full-length Chonburi_2024_209-Ma were C-to-T substitutions. The frequencies of C-to-T substitutions in these 2 genes were 6.38% and 12.14%, respectively, which were higher than those observed in other genes with C-to-T substitutions (< 1%) (Supplementary Table 2). The alignments of these 2 gene sequences were shown in Supplementary Figures 4, 5. The substitutions at nt 34,369 and 35,880 led to early stop codons, Gln93* and Gln41* in MGF_505-2R and MGF_505-3R, respectively. Consequently, the resulting polypeptides were truncated from 526 amino acids to 92 for MGF_505-2R, and from 280 amino acids to 40 for MGF_505-3R. In addition, four genes with premature stop codons were identified. The MGF300-2R and A224L developed early stop codons due to a frameshift mutation resulting from base insertions, meanwhile MGF110-1L and MGF360-11L showed nucleotide substitutions that generated premature stop codons, resulting in truncated polypeptides at Trp197* and Gln22*, respectively.

## 4 Discussion

Genome assemblies based on only short-read data present difficulty in resolving repetitive regions (Treangen and Salzberg, 2011). The presence of ITRs at both ends of ASFV genome together with low read depth at terminal sequences make it difficult to obtain a full-length ASFV assembly, as also noted by Chapman et al. (2008). Complete ITRs and hairpin sequences have been resolved in only one genome, BA71V, which utilized sequencing data from a combination of Sanger sequencing technology (Yanez et al., 1995), previously reported ITR sequences (de la Vega et al., 1994), and hairpin loop sequences obtained with a Maxam–Gilbert technique (Gonzalez et al., 1986). Here, we report another full-length ASFV genome with complete ITR and hairpin sequences. Consistent with the BA71V and HK_NT_202103 genomes, our genome contains four ORFs encoding two genes within both ITRs. These findings demonstrate the significance of a complete ASFV genome assembly in providing valuable insights into ASFV terminal region structure.

The hairpin loop sequences consist of 37 AT-rich nucleotides, as reported in BA71V (Gonzalez et al., 1986; Yanez et al., 1995). However, the nucleotide sequences are not identical. The secondary structure of the hairpin loop with the minimal free energy shows 3 single-stranded loops and 2 stems within the 37-nucleotide sequence (Figure 3). This structure is different from the structure reported in Gonzalez et al. (1986), plausibly because we took adjacent sequences at both sides of the hairpin loop into account in the evaluation of hairpin loop formation. The negative free-energy value indicates spontaneous formation and higher stability of the loop presented here, compared to that reported by Gonzalez et al. (1986). These secondary structures facilitate DNA folding and circularization, providing essential regulatory elements for viral functions and persistence (Chapman et al., 2008). Understanding these hairpin loop topologies is critical for interpreting the virus’s reproduction kinetics and investigating possible antiviral treatments (Gonzalez et al., 1986; Dixon et al., 2013).

The deletion pattern in RVR of our complete full-length Chonburi_2024_209-MA assembly, when compared to Georgia 2007/1, was also similarly observed in the genome of ASFV strain Ratchaburi_2023_001-MA (GCA_032918185.1) (Thaweerattanasinp et al., 2024), and other ASFV genomes adapted to monkey kidney cells (Chapman et al., 2008; Krug et al., 2015; Mazloum et al., 2021), suggesting that these changes may have provided a selective advantage during the virus adaptation process, which is essentially a selection pressure for less lethal viral strains that can infect monkey kidney cells.

The genetic variation of four genotype II ASFV isolates provides insights into the mutation profile, revealing both shared and unique variants. The shared variants may represent conserved mutations within this lineage, whereas the unique mutations could contribute to strain-specific phenotypic differences or host adaptation mechanisms. The high frequency of C-to-T substitutions in MGF505-2R and MGF505-3R genes may be the result of APOBEC mutagenesis. The cytidine deamination activity of APOBEC/AID protein family, especially APOBEC3, can cause C-to-U alteration in ssDNA during replication, subsequently resulting in C-to-T substitution (Hoopes et al., 2016; Xu et al., 2020). APOBEC3 enzymes typically target cytosines within specific sequence motifs, most commonly 5′-TC-3′ or 5′-CC-3′, which is consistent with the pattern observed in this study. This mechanism is an innate immune response by hosts against viruses via deaminase-dependent hypermutation, which has been reported in several viruses e.g., HIV-1, human herpesviruses, human papillomavirus (Xu et al., 2020; Jonathan and Ikeda, 2023). However, this phenomenon has never been reported in ASFV. This study is the first to suggest that APOBEC mutagenesis may occur in the ASFV genome. Although other ASFV genomes adapted to MA-104 cell culture did not exhibit a similar pattern of substitutions (Thaweerattanasinp et al., 2024), the influence of the cell lines used for ASFV propagation cannot be overlooked. LaRue et al. (2008) reported that humans and non-human primates possess 7 APOBEC3 genes, while pigs have only 2 genes. This might be the reason that underlies the finding of this study. Nonetheless, the cause of this C-to-T hypermutation needs further investigation.

The MGF505-2R and MGF505-3R proteins can counteract the host immune system, especially through the cGAS-STING pathway and ferroptosis (Sunwoo et al., 2024; Niu et al., 2025). The deletion of MGF360/505 gene clusters including MG505-2R and MGF505-3R has been reported to attenuate ASFV virulence (Rathakrishnan et al., 2022; Sunwoo et al., 2024; Vu and McVey, 2024). Krug et al. (2015) also reported that the deletions of MGFs potentially influenced ASFV adaptation to Vero cells. Although there was no deletion of the LVR in our genome, similar attenuation and adaptation to MA-104 cells likely occurred in Chonburi_2024_209-MA as the truncated polypeptides of not only MGF505-2R and MGF505-3R, but also MGF300-2R, MGF110-1L, and MGF360-11L potentially resulted in loss of function. The attenuated, cell-adapted variant may have gained a fitness advantage, enabling it to outcompete other strains and become dominant in the population.

This study represents a significant advance in ASFV genomics as it provides one of the first complete hairpin-to-hairpin genome assemblies. Previous attempts to assemble the ASFV genome using short-read technologies were limited in their ability to resolve the terminal regions (Chapman et al., 2008). Even some long-read based assembly approaches struggled with the complex hairpin structures (Rodriguez et al., 2015; Licheri et al., 2024). Our ability to extract a sufficient amount of native ASFV DNA for Oxford Nanopore sequencing and our awareness of the existence of hairpin structures enabled us to accurately reconstruct the terminal hairpin loops without the artificial duplications that occurred in previous assembly attempts (Thaweerattanasinp et al., 2024). In addition, our assembly strategy reveals the presence of the multigene families near the terminal regions, which are often incompletely assembled or incorrectly annotated in previously published reference genomes. The complete genome structure presented here, including intact hairpin loops, provides a more accurate template for future comparative genomics and functional studies of ASFV. This study also underscores the importance of utilizing long-read sequencing platforms that can sequence native DNA to perform genome assembly, not only for the ASFV, but also for other double-stranded DNA viruses with a terminal cross-bridge sequence, such as poxviruses (Dixon et al., 2013).

## Data Availability

The datasets presented in this study can be found in online repositories. The names of the repository/repositories and accession number(s) can be found below: https://www.ncbi.nlm.nih.gov/, PRJNA1244047.

## Footnotes

1
https://www.unafold.org/mfold/applications/dna-folding-form.php
