# Supplementary material for: Hairpin loop to hairpin loop: a full-length assembly of the ASFV genome using Oxford Nanopore long-read sequencing
Source: Front Microbiol. 2025 Aug 8;16:1615977. doi: 10.3389/fmicb.2025.1615977 (PMC12370676; doi:10.3389/fmicb.2025.1615977)
Supplement: Supplementary file 1 [file Data_Sheet_1.docx]

Supplementary Material

**
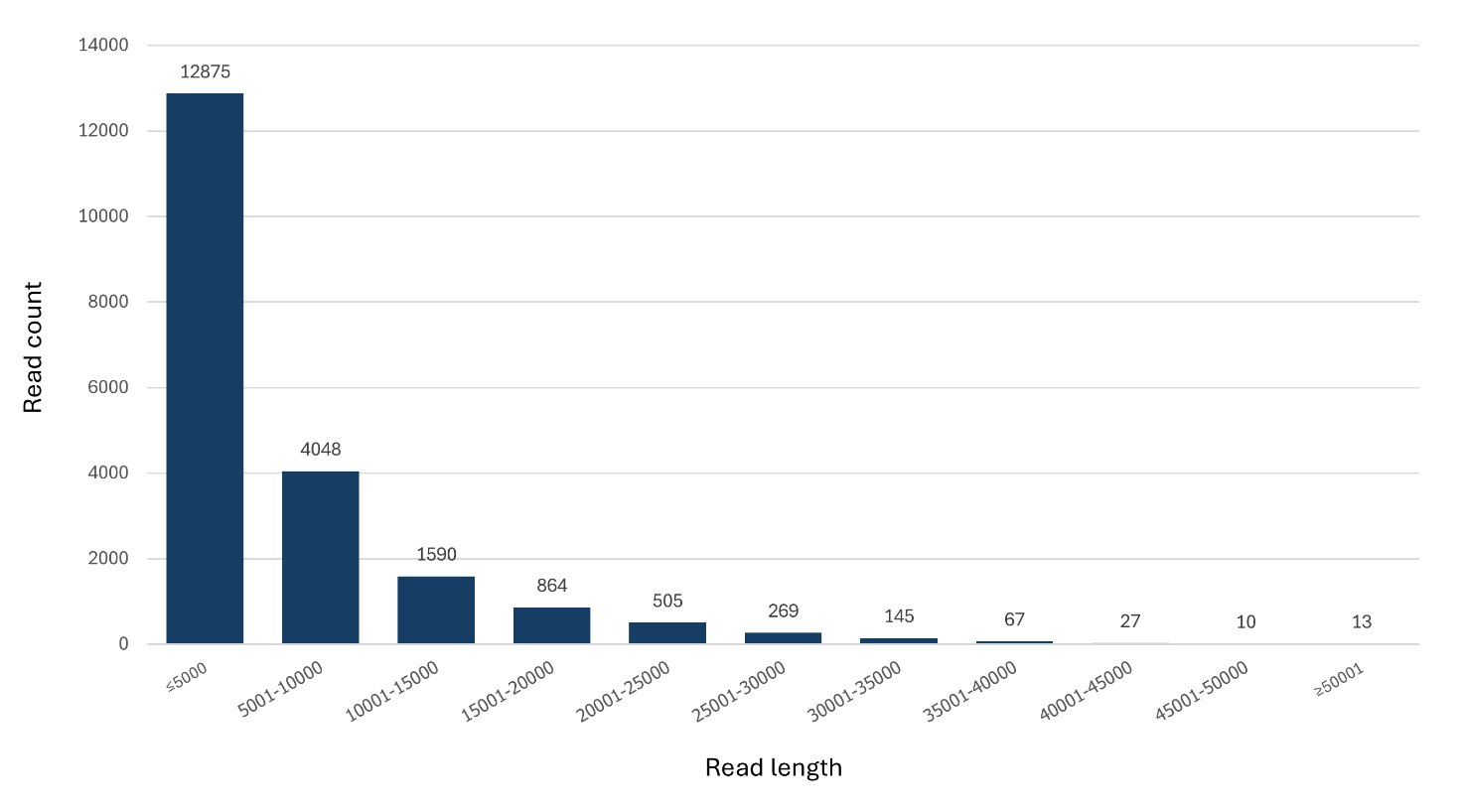
**

**Supplementary Figure 1.** The distribution of Oxford Nanopore read length used for assembly. Reads smaller than 1000 bases were excluded.


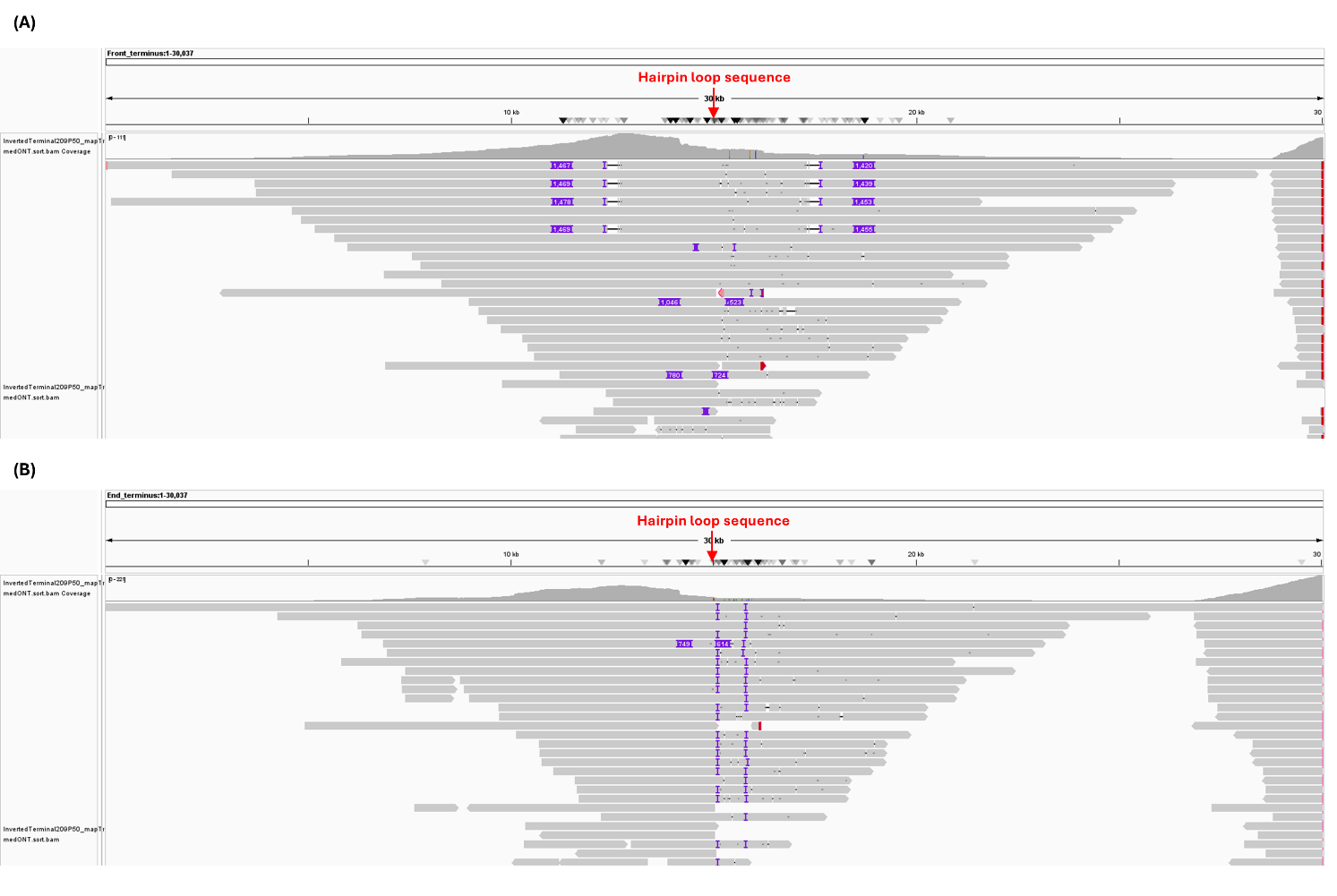


**Supplementary Figure 2.** Oxford Nanopore reads mapped on front-terminal (A) and back-terminal (B) duplicated reverse complementary sequences with hairpin loop sequence in the middle. Reads spanned across hairpin loop sequence displays symmetrical length on left and right sides.


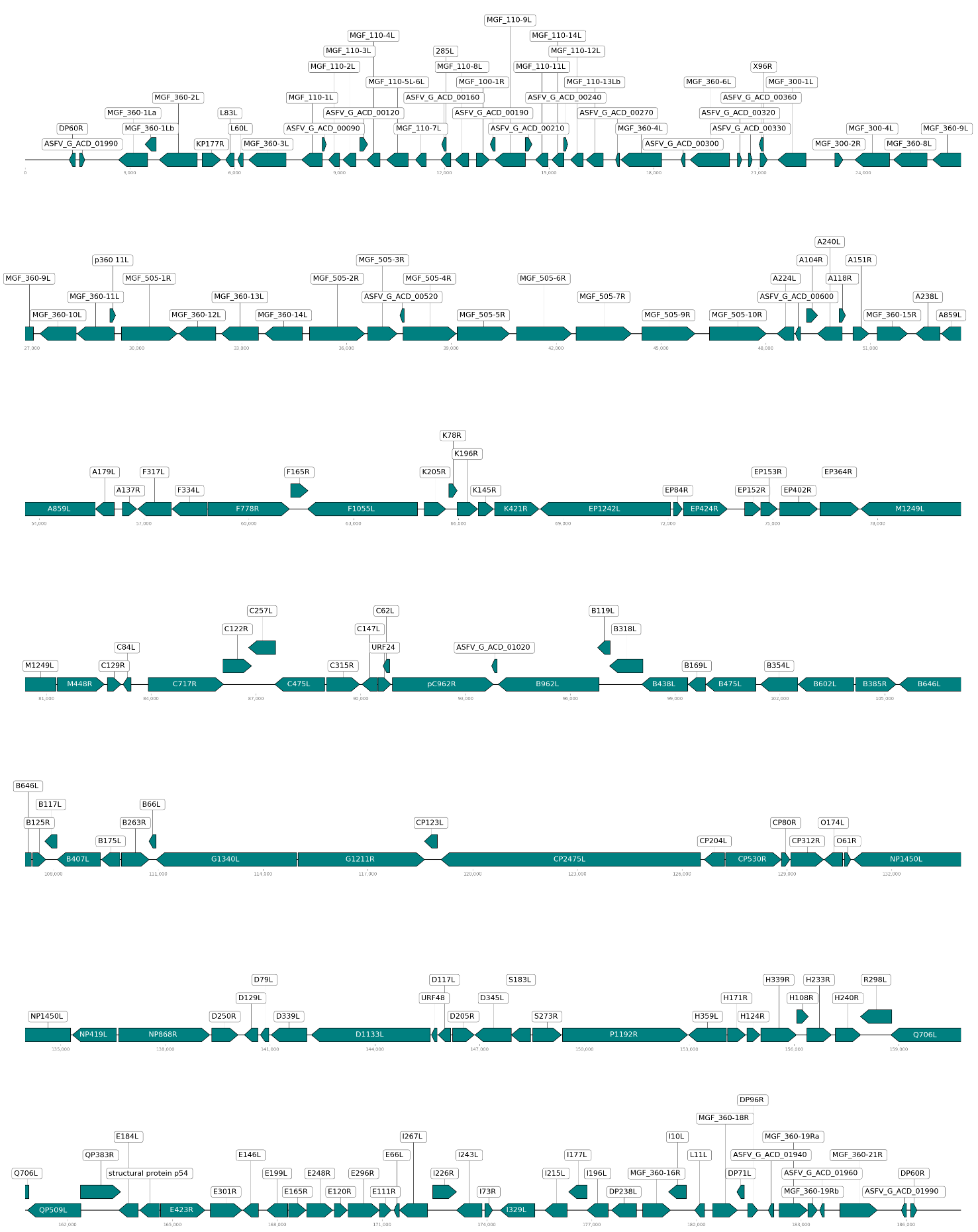


**Supplementary Figure 3.** Gene arrangements of the complete full-length Chonburi_2024_209-MA assembly containing 186 ORFs.


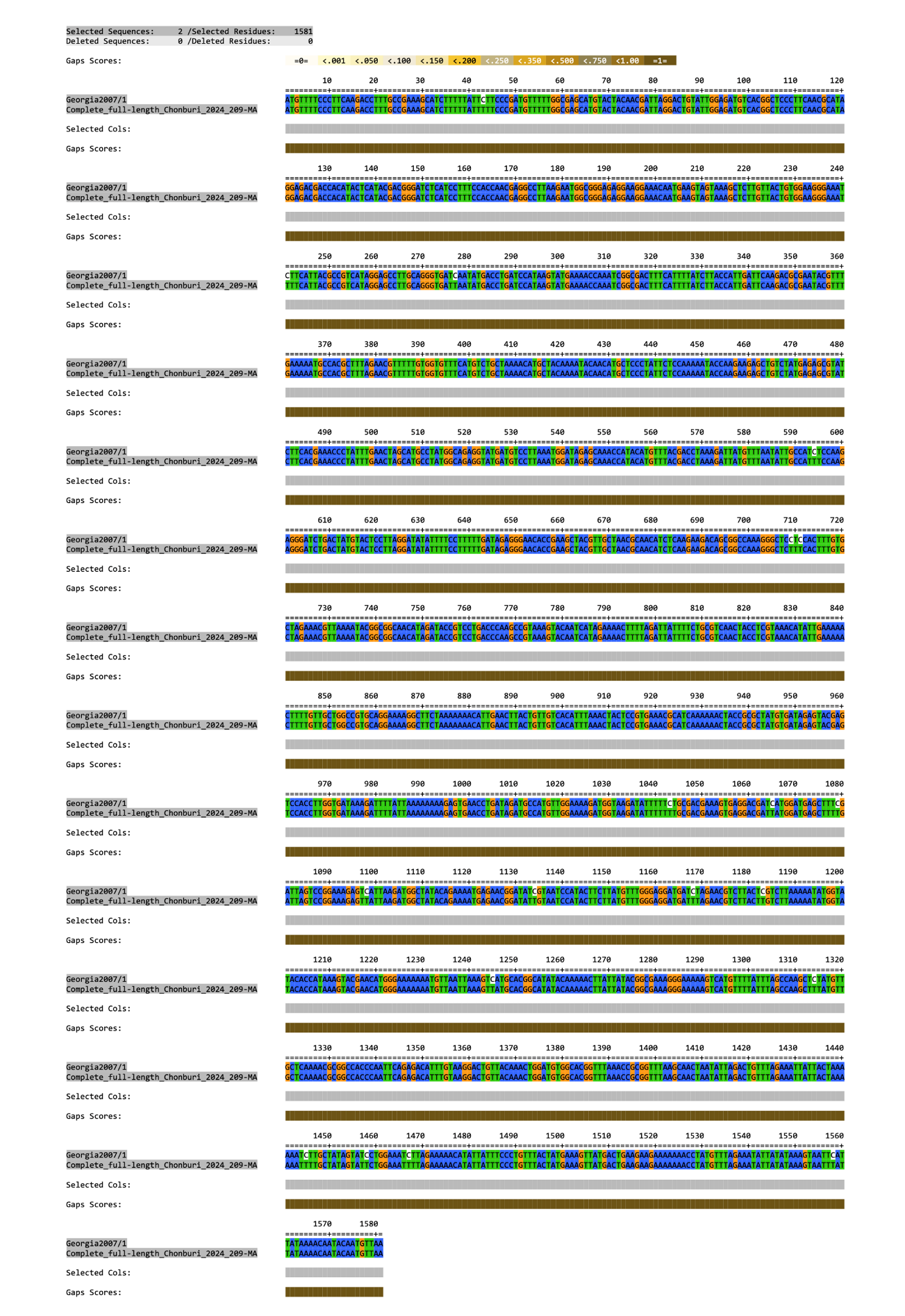


**Supplementary Figure 4.** The alignment of MGF505-2R sequences between Georgia2007/1 and the complete full-length Chonburi_2024_209-MA.


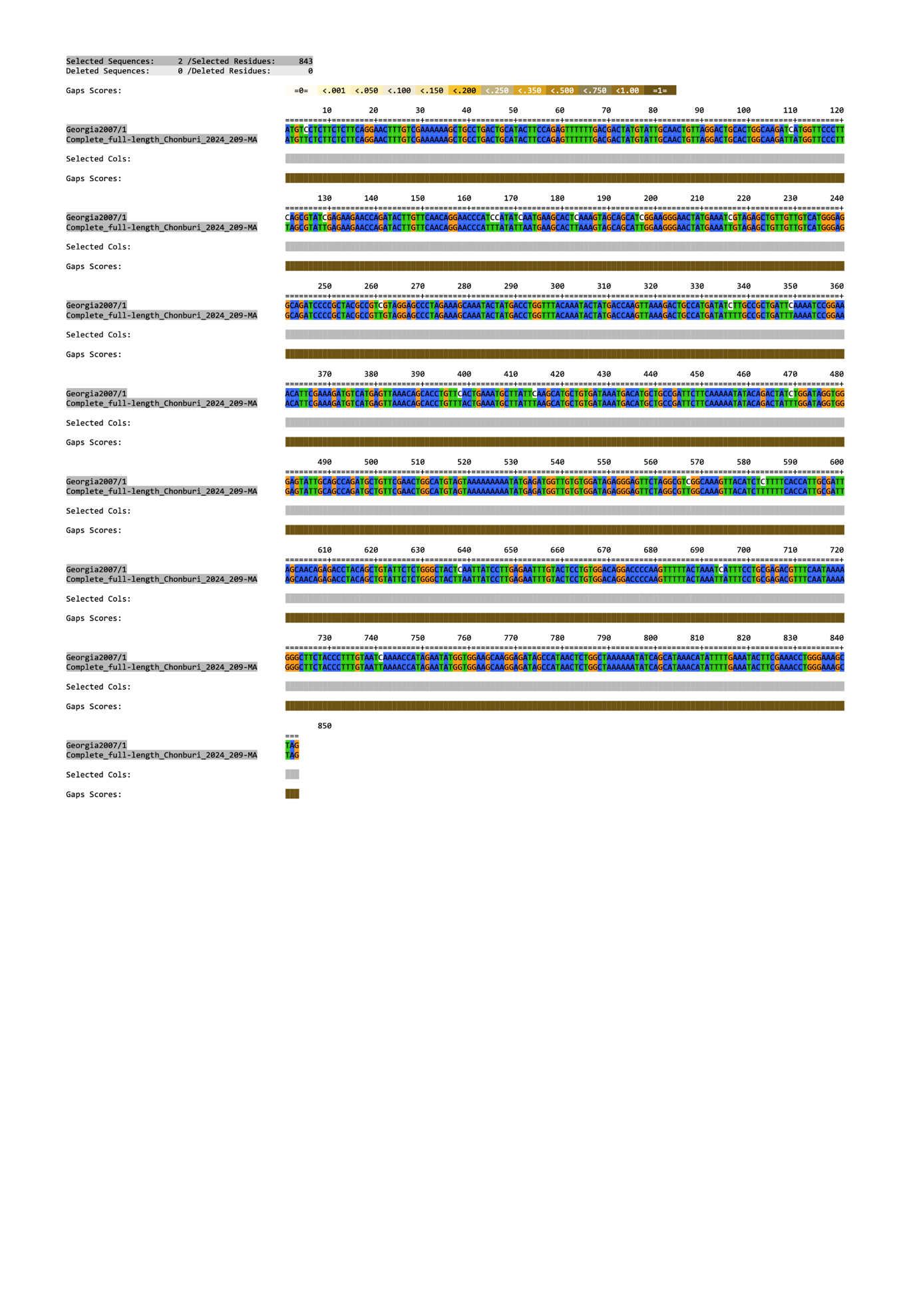


**Supplementary Figure 5.** The alignment of MGF505-3R sequences between Georgia2007/1 and the complete full-length Chonburi_2024_209-MA.
